# An integrated teaching model combining PBL and medical humanities education for pathology laboratory teaching: a randomized controlled study

**DOI:** 10.1186/s12909-026-09701-x

**Published:** 2026-06-15

**Authors:** Sufen Wang, Jun Shen, Banghong Qiang, Honghai Xu, Xiaomei Huang, Lizheng Wang

**Affiliations:** 1 Department of Pathology, The First Affiliated Hospital of Wannan Medical University (Yijishan Hospital of Wannan Medical University), Wuhu City, Anhui Province 241001 People’s Republic of China; 2 Department of Neurosurgery, The First Affiliated Hospital of Wannan Medical University (Yijishan Hospital of Wannan Medical University), Wuhu City, Anhui Province 241001 People’s Republic of China; 3 Department of Ultrasound Medicine, Wuhu Second People’s Hospital, Wuhu City, Anhui Province 241001 People’s Republic of China; 4 Department of Pathology, The First Affiliated Hospital of Anhui Medical University, Hefei City, Anhui Province 230022 People’s Republic of China

**Keywords:** Medical humanities, Problem-based learning, Pathology education, Randomized controlled trial

## Abstract

**Objective:**

To evaluate the educational effectiveness of an integrated teaching model combining problem-based learning (PBL) and medical humanities education for pathology laboratory teaching.

**Methods:**

This randomized controlled study was conducted from September 2025 to January 2026 among undergraduate anesthesiology students enrolled in a pathology laboratory course at Wannan Medical University. Four classes were randomly assigned at the class level to either the experimental group, which received the integrated PBL and medical humanities teaching model (*n* = 56), or the control group, which received a traditional LBL teaching model (*n* = 59). Following the instructional intervention, theoretical examination scores, microscopic image interpretation scores, questionnaire-based teaching effectiveness outcomes, and overall student satisfaction were compared between groups.

**Results:**

Both groups showed significant improvements in theoretical examination scores after the intervention compared with baseline (both *P* < 0.001). After adjustment for class-level clustering using linear mixed-effects models, no significant between-group difference was observed in post-intervention theoretical examination scores (*P* = 0.122), while microscopic image interpretation scores showed a borderline between-group difference favoring the experimental group (*P* = 0.061). Compared with the control group, students in the experimental group achieved significantly higher scores in learning motivation, self-directed learning ability, and communication and expression skills (all *P* < 0.05). Teamwork ability showed a borderline between-group difference (*P* = 0.058), whereas differences in professional ethics awareness and understanding of healthcare-related social responsibility were not statistically significant. Overall teaching satisfaction was significantly higher in the experimental group than in the control group (*P* = 0.002).

**Conclusion:**

Compared with traditional LBL teaching, the integrated teaching model combining PBL and medical humanities education was associated with more favorable questionnaire-based outcomes in learning motivation, self-directed learning ability, and communication and expression skills, as well as higher student satisfaction. This integrated instructional approach may provide a useful reference for the ongoing reform and improvement of pathology laboratory education.

**Supplementary Information:**

The online version contains supplementary material available at 10.1186/s12909-026-09701-x.

## Background

Pathology serves as a bridge between basic and clinical medicine, playing a crucial role in cultivating medical students’ understanding of disease pathogenesis, diagnosis, differential diagnosis, and clinical management [1, 2]. As an essential component of pathology education, laboratory teaching facilitates the translation of theoretical knowledge into practical skills and is indispensable for helping students understand disease progression and morphological diagnostic features. However, most medical schools still primarily use a teacher-centered demonstration-observation approach in pathology laboratory teaching. In this model, instructors typically deliver lectures and demonstrate key microscopic features, after which students independently observe histopathological slides under the microscope. Nevertheless, the overall process remains predominantly teacher-directed, with limited active engagement from students. While this teaching model demonstrates some efficiency in knowledge transfer and instructional organization, it also exhibits notable limitations in educational practice.

Traditional lecture-based learning (LBL) in pathology laboratory teaching often emphasizes knowledge transmission and standardized answers, which may lead to passive learning, low student participation, and insufficient stimulation of learning motivation and critical thinking [3, 4]. Furthermore, this teaching model limits the development of students’ clinical reasoning, communication, and teamwork abilities, and often neglects the integration of medical humanities. As medical education shifts from a “biomedical model” to a “biopsychosocial model,” purely knowledge- and technology-oriented teaching methods are no longer adequate to meet the evolving demands of modern medical education. In this context, there is a growing need for teaching approaches that not only enhance students’ cognitive learning but also foster humanistic competencies and professional development. Medical humanities education, emphasizing empathy, ethical awareness, communication skills, and social responsibility, has become an increasingly important component of contemporary medical education. Research indicates that medical humanities programs can improve empathy and related humanistic competencies among medical students and health professionals [5, 6]. Meanwhile, student-centered teaching methods such as problem-based learning (PBL) are widely recognized for promoting active learning, critical thinking, teamwork, and the comprehensive application of knowledge [7–9]. An integrated teaching model combining PBL and medical humanities education may encourage students to consider not only the disease itself but also the patient experience and broader social context, thereby facilitating the parallel development of medical knowledge and professional values.

Although both medical humanities education and PBL have recognized advantages in medical education, limited evidence exists regarding the effectiveness of integrated teaching models combining PBL and medical humanities education in pathology laboratory teaching, particularly in undergraduate medical education. Therefore, this study aims to evaluate the educational effectiveness of an integrated teaching model in pathology laboratory teaching, with the goal of providing empirical evidence to inform and support ongoing educational reform in pathology laboratory curricula.

## Materials and methods

### Students

This study was designed as a prospective cluster randomized controlled trial. Participants were second-year undergraduate students majoring in anesthesiology at Wannan Medical University who were enrolled in a pathology laboratory course. A total of four classes were included, with approximately 28–30 students in each class. These classes were randomly assigned at the class level to either an experimental group or a control group. The experimental group received an integrated teaching model combining PBL and medical humanities education, whereas the control group received a teacher-centered demonstration–observation experimental teaching method (LBL). All students had completed the basic theoretical course in digestive system pathology prior to participation in the study. The baseline characteristics of students in the two groups are shown in Table 1. There were no significant differences between the two groups in sex distribution or age. After accounting for class-level clustering using a linear mixed-effects model, no significant difference was observed in pre-intervention theoretical knowledge scores between the two groups. This study was approved by the Ethics Committee of the First Affiliated Hospital of Wannan Medical University (Yijishan Hospital of Wannan Medical University).

**Table 1:** Baseline characteristics and pre-intervention theoretical scores of students in the experimental and control groups

Variables	Control group (*n* = 59)	Experimental group (*n* = 56)	Statistic	*P* value
Age (years, mean ± SD)	19.458 ± 0.625	19.429 ± 0.710	*t* = 0.233	0.816
Sex, n (%)			*χ²* = 0.017	0.896
Male	26 (44.1%)	24 (42.9%)		
Female	33 (55.9%)	32 (57.1%)		
Pre-intervention theoretical score (adjusted mean, 95% CI)	65.949 (65.010–66.888)	66.625 (65.661–67.589)	*F* = 0.990	0.322

Values are presented as mean ± SD or n (%). Age was compared using an independent-samples t-test, sex distribution was compared using the χ² test, and pre-intervention theoretical examination scores were analyzed using a linear mixed-effects model with teaching group as a fixed effect and class as a random effect to account for class-level clustering.

### Study design

Participants were randomized at the class (cluster) level into either an experimental group, which received the integrated PBL and medical humanities teaching model (*n* = 56), or a control group, which received a traditional teacher-centered demonstration–observation teaching model (LBL) (*n* = 59). Prior to the intervention, all instructors involved in the study underwent standardized training to ensure consistency in teaching content, instructional procedures, facilitation strategies, and evaluation criteria. To minimize potential bias, the same teaching content, duration, and assessment criteria were applied to both groups. Randomization was performed using a computer-generated sequence by an independent researcher.

### Teaching interventions

#### Experimental group instructional design

**Fig. 1:**
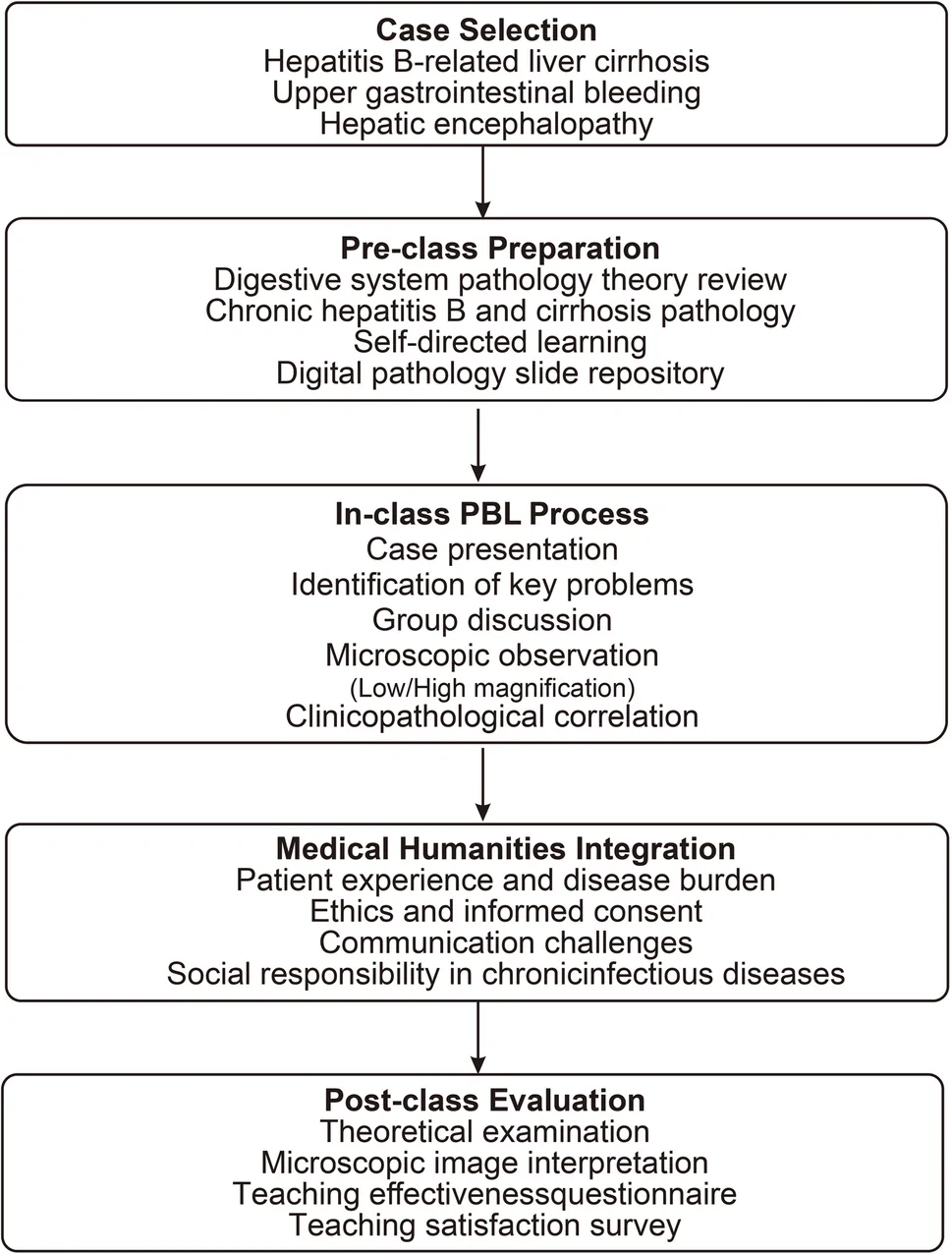
Framework of the integrated PBL and medical humanities teaching model in pathology laboratory education

Students in the experimental group received pathology laboratory instruction based on an integrated PBL and medical humanities teaching model. Teaching was organized around a single representative clinical case of hepatitis B-related liver cirrhosis complicated by upper gastrointestinal bleeding and hepatic encephalopathy, which served as the core instructional context throughout the intervention (Fig. 1).

At the beginning of the instructional process, students were provided with a structured clinical case and worked in small groups to identify key clinical and pathological problems, formulate preliminary diagnostic hypotheses, and define learning objectives related to pathological mechanisms, clinicopathological correlations, differential diagnosis, treatment strategies, and prognosis.

Students were required to integrate prior theoretical knowledge with self-directed learning outside the classroom. Guided by the learning objectives, they reviewed key pathological features of chronic hepatitis B-related cirrhosis, including hepatocellular injury, fibrous septa formation, and pseudolobule development, and explored gross and microscopic morphologic features using the institutional digital pathology slide repository.

During the laboratory session, students performed microscopic examination of liver cirrhosis slides, focusing on systematic morphological description under low- and high-power magnification and pathological interpretation. Group discussions were conducted to integrate microscopic findings with clinical data and disease progression, with instructors acting as facilitators rather than lecturers.

Medical humanities education was embedded throughout the PBL process. Humanistic elements arising from the case, including long-term disease burden, ethical considerations, communication challenges, stigma associated with chronic infectious diseases, and physicians’ social responsibility, were incorporated into group discussions and reflections.

At the conclusion of the session, each group presented a structured summary of pathological findings, clinicopathological correlations, and humanistic considerations. Instructors provided feedback to ensure alignment with learning objectives.

#### Control group instructional design

Students in the control group received pathology laboratory instruction using a traditional LBL teaching model. The instructional content was centered on the same hepatitis B–related liver cirrhosis case to ensure consistency between groups.

During the laboratory session, the instructor delivered a didactic presentation explaining the key microscopic features and diagnostic characteristics of liver cirrhosis. Students then independently observed histopathological slides under light microscopy and identified relevant morphologic features based on the instructional content. Teaching was primarily instructor-centered, with limited opportunities for student interaction, discussion, or collaborative learning.

#### Teaching evaluation and data collection

Teaching effectiveness was evaluated after completion of the instructional intervention using a combination of objective assessments and questionnaire-based surveys. 

#### Theoretical knowledge assessment

Students completed a post-intervention theoretical examination (total score: 100). The examination assessed students’ understanding of core concepts in digestive system pathology, particularly pathological changes associated with hepatitis B–related liver cirrhosis. 

#### Microscopic image interpretation assessment

A microscopic image interpretation test (total score: 100) was administered to evaluate students’ abilities in morphologic observation, microscopic description, and basic pathological interpretation under both low- and high-power magnification. 

#### Evaluation of teaching effectiveness

Teaching effectiveness was assessed using a self-developed structured questionnaire developed according to the study objectives and relevant literature. The questionnaire comprised 12 items across six domains: learning motivation, self-directed learning ability, communication and expression skills, teamwork ability, professional ethics awareness, and understanding of healthcare-related social responsibility. Each item was rated on a 5-point Likert scale (see Supplementary File S1). The questionnaire demonstrated good internal consistency (Cronbach’s alpha = 0.89). 

#### Teaching satisfaction survey

Teaching satisfaction was assessed using a single-item question in which students rated their overall satisfaction as very satisfied, satisfied, neutral, or dissatisfied. Overall satisfaction was defined as the proportion of students reporting either “very satisfied” or “satisfied.”

### Statistical analysis

Statistical analyses were performed using SPSS version 26.0 (IBM Corp., Armonk, NY, USA). Continuous variables are presented as mean ± standard deviation (SD) or adjusted means with 95% confidence intervals (CIs), as appropriate, and categorical variables are expressed as counts and percentages. Baseline age was compared using an independent-samples t-test, and sex distribution was compared using the χ² test. Within-group comparisons of pre- and post-intervention theoretical examination scores were performed using paired-samples t-tests.

Because randomization was conducted at the class level, linear mixed-effects models were used for between-group comparisons to account for class-level clustering. In these models, teaching group was included as a fixed effect and class was included as a random effect. Post-intervention theoretical examination scores, microscopic image interpretation scores, and questionnaire-based outcomes were analyzed using linear mixed-effects models. All statistical tests were two-tailed, and a P value < 0.05 was considered statistically significant.

## Results

### Comparison of students’ theoretical and microscopic image interpretation assessment scores

Within-group pre- and post-intervention comparisons are presented in Table 2, whereas between-group post-intervention comparisons are summarized in Table 3. Compared with baseline levels, both the control and experimental groups demonstrated significant improvements in theoretical examination scores after the intervention (paired t-test, both *p* < 0.05) (Table 2). After adjustment for class-level clustering using linear mixed-effects models, no statistically significant difference was observed between groups in post-intervention theoretical examination scores (adjusted mean difference = 2.86, 95% CI: -1.93 to 7.65, *P* = 0.122). Microscopic image interpretation scores were higher in the experimental group, with a borderline between-group difference after adjustment for class-level clustering (adjusted mean difference = 5.72, 95% CI: -0.63 to 12.07, *P* = 0.061) (Table 3).

**Table 2:** Within-group comparison of pre- and post-intervention theoretical examination scores

Variables	Control group (*n* = 59)	Experimental group (*n* = 56)
Pre-intervention theoretical score	65.949 ± 3.901	66.625 ± 3.344
Post-intervention theoretical score	76.000 ± 4.102	78.839 ± 4.343
*t*	13.637	16.674
*P value*	< 0.001	< 0.001

Within-groupcomparisons of pre- and post-intervention theoretical examinationscores were performed using paired-samples t-tests

**Table 3:** Between-group comparison of post-intervention theoretical examination and microscopic image interpretation scores using linear mixed-effects models

Variables	Control group adjusted mean (95% CI)	Experimental group adjusted mean (95% CI)	*F* statistic	*P* value
Post-intervention theoretical score	75.999 (72.542–79.455)	78.858 (75.537–82.178)	6.961	0.122
Microscopic image interpretation score	82.357 (77.831–86.882)	88.077 (83.625–92.529)	14.919	0.061

Values are adjusted means (95% confidence intervals) estimated from linear mixed-effects models with teaching group as a fixed effect and class as a random effect to account for class-level clustering. *P *values were derived from linear mixed-effects models

### Comparison of students’ evaluation of teaching effectiveness based on questionnaire survey

**Fig. 2:**
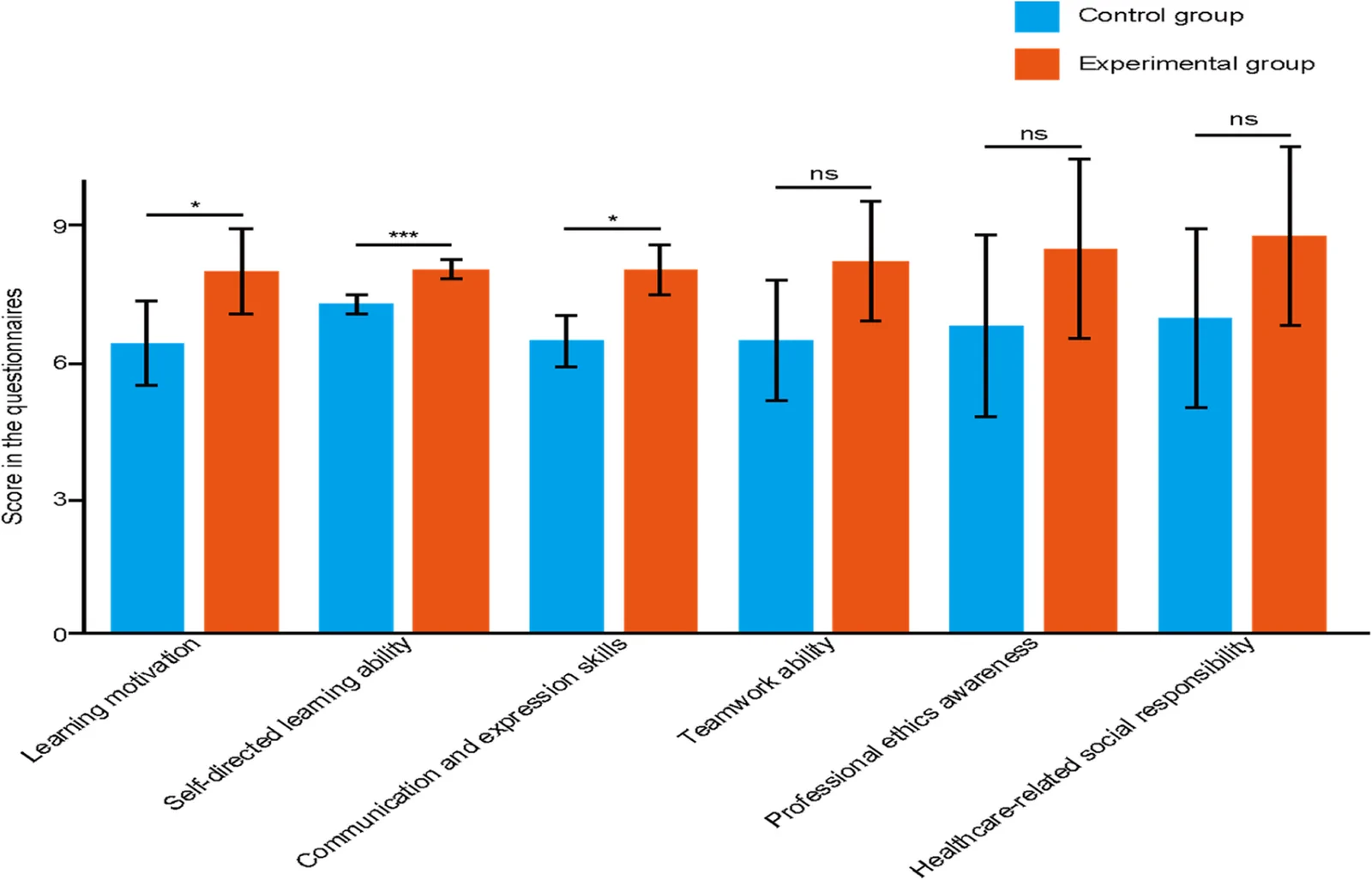
Comparison of teaching effectiveness outcomes between the experimental and control groups. Bars represent adjusted means estimated from linear mixed-effects models, and error bars indicate 95% confidence intervals. *P < 0.05, ***P < 0.001

After the intervention, a questionnaire survey was administered to assess teaching effectiveness in both groups. After adjustment for class-level clustering using linear mixed-effects models, the experimental group showed significantly higher scores than the control group in learning motivation, self-directed learning ability, and communication and expression skills (all *P* < 0.05). Teamwork ability showed a borderline between-group difference (*P* = 0.058). Although professional ethics awareness and understanding of healthcare-related social responsibility scores were higher in the experimental group, these differences did not reach statistical significance after adjustment for class-level clustering (Table 4). The comparison of teaching effectiveness between the experimental and control groups is illustrated in Fig. 2.

**Table 4:** Comparison of questionnaire-based teaching effectiveness outcomes between groups using linear mixed-effects models

Variables	Control group adjusted mean (95% CI)	Experimental group adjusted mean (95% CI)	*F﻿*	*P* value
Learning motivation	6.369 (5.425–7.313)	7.951 (7.018–8.884)	26.117	0.036
Self-directed learning ability	7.237 (7.025–7.450)	8.000 (7.782–8.218)	24.630	< 0.001
Communication and expression skills	6.422 (5.849–6.995)	7.983 (7.426–8.541)	68.564	0.014
Teamwork ability	6.437 (5.107–7.767)	8.171 (6.854–9.489)	15.955	0.058
Professional ethics awareness	6.755 (4.764–8.745)	8.444 (6.460–10.428)	6.683	0.123
Understanding of healthcare-related social responsibility	6.922 (4.957–8.886)	8.732 (6.772–10.693)	7.859	0.107

Values are adjusted means (95% confidence intervals) estimated from linear mixed-effects models with teaching group as a fixed effect and class as a random effect to account for class-level clustering. *P* values were derived from linear mixed-effects models

### Comparison of students’ teaching satisfaction based on questionnaire survey

As shown in Table 5, overall teaching satisfaction differed significantly between the experimental and control groups (χ² = 15.356, *P* = 0.002). Students in the experimental group reported higher overall teaching satisfaction than those in the control group. Specifically, the proportion of students who were very satisfied was higher in the experimental group (55.4% vs. 20.3%), whereas the proportions reporting average satisfaction (17.9% vs. 28.8%) or dissatisfaction (5.4% vs. 13.6%) were lower than those in the control group.

**Table 5:** Comparison of teaching satisfaction between the experimental and control groups

Variables	Control group (*n* = 59)	Experimental group (*n* = 56)	*χ²*	*P* value
Overall satisfaction [n(%)]			15.356	0.002
Dissatisfied	8 (13.6%)	3 (5.4%)		
Average	17 (28.8%)	10 (17.9%)		
Quite satisfied	22 (37.3%)	12 (21.4%)		
Very satisfied	12 (20.3%)	31 (55.4%)		

Values are presented as n (%). Differences in satisfaction distribution between groups were compared using the χ² test

## Discussion

In this randomized controlled study, we evaluated the educational effectiveness of an integrated teaching model combining PBL and medical humanities education in pathology laboratory teaching using a representative clinical case of hepatitis B–related liver cirrhosis complicated by upper gastrointestinal bleeding and hepatic encephalopathy. Compared with traditional LBL teaching, the integrated instructional model showed less robust differences in post-intervention theoretical examination and microscopic image interpretation outcomes after adjustment for class-level clustering. In contrast, the integrated teaching model was associated with more favorable questionnaire-based outcomes in learning motivation, self-directed learning ability, and communication and expression skills.

These findings are generally consistent with previous studies suggesting that PBL-based educational approaches may enhance student engagement, active participation, and self-directed learning compared with traditional LBL approaches [10–12].

The selected case provided a clinically and pathologically complex learning scenario with substantial educational value. The patient’s long-standing chronic liver disease, acute upper gastrointestinal bleeding, and neuropsychiatric manifestations of hepatic encephalopathy required students to integrate hepatic pathological changes, such as hepatocellular degeneration and necrosis, collapse of the hepatic framework, and pseudolobule formation with disease progression and end-stage complications. This process fostered morphology-based clinicopathological reasoning and encouraged students to move beyond fragmented knowledge toward a coherent understanding of disease evolution. Such levels of case complexity are well suited to case-based and discussion-oriented educational models, which may support learners in integrating information across domains and engaging in analytical reasoning processes [13].

Teaching further emphasized the pathological continuum of chronic hepatitis B–related liver disease, guiding students from initial hepatocyte injury and necrosis to reticulin framework collapse, fibrous tissue proliferation, and pseudolobule formation. Through independent review of theoretical content and digital pathology resources, students identified characteristic morphologic features within pseudolobules and interlobular fibrous septa. This case-centered and problem-driven instructional approach may have facilitated students’ integration of theoretical knowledge with the understanding of pathological morphologic features and clinical reasoning processes. These findings are broadly consistent with constructivist learning theory, which emphasizes active knowledge construction through contextualized and clinically relevant problems [14, 15].

Beyond cognitive outcomes, students in the experimental group tended to report higher questionnaire-based scores in domains related to learning motivation, self-directed learning ability, communication and expression skills, teamwork ability, professional ethics awareness, and understanding of healthcare-related social responsibility. Some of these questionnaire-based outcomes remained statistically significant after adjustment for class-level clustering, whereas others demonstrated similar trends favoring the experimental group without reaching conventional statistical significance. These findings suggest that the integrated teaching model may be associated with perceived improvements in learning engagement and certain humanistic competency-related domains. However, because these outcomes were assessed using a self-developed questionnaire that has not undergone external validation, the questionnaire-based findings should be interpreted with caution.

The selected clinical case incorporated complex medical and humanistic issues, including chronic illness burden, patient suffering, communication challenges, and ethical considerations, which may have provided opportunities for students to reflect on professional responsibility and social accountability. Previous studies have similarly suggested that medical humanities education may support empathy, ethical awareness, and reflective capacity among medical students [16–18].

Higher teaching satisfaction among students in the experimental group may reflect the educational value of the integrated teaching model. Active participation, small-group discussion, and engagement with authentic clinical and humanistic issues have been associated with increased learner engagement and perceived relevance of medical education [19, 20]. In contrast, traditional teacher-centered demonstration–observation methods, while effective for standardized knowledge delivery, may provide fewer opportunities for interaction and reflective learning [21].

Nevertheless, several challenges associated with implementing the integrated PBL and medical humanities teaching model should be acknowledged. This integrated teaching model places higher demands on instructors’ facilitation skills and humanistic competence, requires greater student engagement and preparation, and often necessitates additional instructional time and organizational resources. Similar concerns regarding faculty workload, curricular complexity, and resource intensity have been reported in previous evaluations of PBL-based curricula [22, 23]. Moreover, inappropriate case selection or insufficient instructional guidance may result in superficial discussions of humanistic issues that are insufficiently connected to the intended learning objectives.

It should be noted that the present study compared an integrated instructional model combining PBL and medical humanities education with a traditional LBL approach. Therefore, the observed findings likely reflect the overall educational effects of the integrated teaching model rather than the isolated contribution of any single component. Future studies comparing PBL alone with integrated PBL and medical humanities teaching models may help further clarify the potential contribution of different instructional components.

This study also has several limitations. First, although the questionnaire demonstrated good internal consistency, it was self-developed and has not undergone rigorous external validation. Therefore, the questionnaire-based findings, particularly those related to humanistic competency-related domains, should be interpreted with caution because they may be subject to self-reporting and measurement bias. Second, because randomization was performed at the class level and only a limited number of clusters were included, the statistical power of the mixed-effects model analyses may have been constrained, which could have contributed to the attenuation of statistical significance in some outcomes after adjustment for class-level clustering. In addition, this study was conducted at a single center with a relatively small sample size and only evaluated short-term educational outcomes. Future studies with larger samples, more clusters, and longer follow-up periods are warranted.

## Conclusion

In conclusion, compared with traditional LBL teaching, the integrated teaching model combining PBL and medical humanities education was associated with more favorable questionnaire-based learning outcomes and higher student satisfaction. These findings suggest that this integrated instructional approach may provide a useful reference for the ongoing reform and improvement of pathology laboratory education.

## Supplementary Information

Supplementary Material 1.

## Data Availability

All data generated or analyzed during this study are included in this published article. No additional datasets were generated or analyzed.

## Abbreviations

PBL: Problem-based learning
LBL: Lecture-based learning

## References

[1] Xu C, Li Y, Chen P, Pan M, Bu X. A survey on the attitudes of Chinese medical students towards current pathology education. BMC Med Educ. 2020;20(1):259. 32771019 10.1186/s12909-020-02167-5 PMC7414265

[2] Cai L, Li YL, Hu XY, Li R. Implementation of flipped classroom combined with case-based learning: A promising and effective teaching modality in undergraduate pathology education. Medicine. 2022;101(5):e28782. 35119043 10.1097/MD.0000000000028782 PMC8812661

[3] Zhan HQ, Zhang XX, Qin R, Fei J, Dong GY, Hao JH. Application of integrated problem-based learning combined with lecture-based classroom teaching in undergraduate medical education: An effective teaching model in a Medical School in China. Medicine. 2023;102(34):e34792. 37653783 10.1097/MD.0000000000034792 PMC10470717

[4] Xu L, Lin C, Hou C, Li S. The combined BOPPPS and CBL teaching method enhances students’ learning in pathology experimental course. Adv Physiol Educ. 2025;49(2):526–34. 40171934 10.1152/advan.00154.2024

[5] Zhang X, Pang HF, Duan Z. Educational efficacy of medical humanities in empathy of medical students and healthcare professionals: a systematic review and meta-analysis. BMC Med Educ. 2023;23(1):925. 38057775 10.1186/s12909-023-04932-8 PMC10698992

[6] Engebretsen E, Fraas Henrichsen G, Ødemark J. Towards a translational medical humanities: introducing the cultural crossings of care. Med Humanit. 2020;46(2):e2. 32341131 10.1136/medhum-2019-011751 PMC7402465

[7] de Andrade Gomes J, Braga LAM, Cabral BP, Lopes RM, Mota FB. Problem-Based Learning in Medical Education: A Global Research Landscape of the Last Ten Years (2013–2022). Med Sci Educ. 2024;34(3):551–60. 38887406 10.1007/s40670-024-02003-1 PMC11180071

[8] Ge WL, Zhu XY, Lin JB, Jiang JJ, Li T, Lu YF, et al. Critical thinking and clinical skills by problem-based learning educational methods: an umbrella systematic review. BMC Med Educ. 2025;25(1):455. 40155947 10.1186/s12909-025-06951-z PMC11951611

[9] Fang Y, Chen J, He P. Problem-based learning for functional otolaryngology in postgraduate education. BMC Med Educ. 2025;26(1):155. 41449379 10.1186/s12909-025-08473-0 PMC12853633

[10] Epm SB, Karuppiah Y, Bhuvaneswari K. Influence of Problem-based Learning Method on Learning Outcomes in Medical Curriculum. J Assoc Phys India. 2025;73(8):32–4. 10.59556/japi.73.1079 40955883

[11] Li F, Luo J, Zhang H. The Application of Problem-Based Learning Combined With Case-Based Learning in EEG Teaching. J Med Educ Curric Dev. 2024;11:23821205241252277. 38711831 10.1177/23821205241252277 PMC11072060

[12] Xiong X, Xu J, Luo M, Niu D, Bi Q, Wang Z, et al. Efficacy of problem-based learning combined with case-based learning versus lecture-based learning in orthopedic education: a systematic review and meta-analysis. BMC Med Educ. 2025;25(1):1357. 41053695 10.1186/s12909-025-07741-3 PMC12502553

[13] Lim JJ, Veasuvalingam B. Does online case-based learning foster clinical reasoning skills? A mixed-methods study. Future Healthc J. 2025;12(1):100210. 39654610 10.1016/j.fhj.2024.100210 PMC11625323

[14] Chao X, Li X. The application of the conceive-design-implement-operate combined with the problem-based-learning teaching method in an 8-year rotation system for obstetrics students in China. BMC Med Educ. 2025;25(1):1077. 40682032 10.1186/s12909-025-07646-1 PMC12272961

[15] Sarmiento-Rojas J, Aya-Parra PA, Perdomo OJ. Proposal of Design and Innovation in the Creation of the Internet of Medical Things Based on the CDIO Model through the Methodology of Problem-Based Learning. Sensors. 2022;22(22):8979. 10.3390/s22228979 PMC9697009 36433574

[16] Hong Y, Song C, Jiang Z, Zhang W. Mapping the Landscape of Medical Humanities Education: Trends and Insights. J Eval Clin Pract. 2025;31(6):e14239. 39529465 10.1111/jep.14239

[17] Giannari M, Botis GG, Lazari EC, Thymara E, Kavantzas NG, Lazaris AC. Restoring Empathy in Medical Education: The Measurable Impact of a Humanities-Based Course on Empathy. J Med Educ Curric Dev. 2025;12:23821205251405320. 41409441 10.1177/23821205251405320 PMC12705950

[18] Zhai H, Xue J, Wu H, Liao S, Lavender C, Lv Y, et al. A national perspective: integrating medical humanities to address burnout and stress in Chinese medical education. BMC Med Educ. 2025;25(1):304. 40001154 10.1186/s12909-025-06875-8 PMC11863532

[19] Isaac M. Role of humanities in modern medical education. Curr Opin Psychiatry. 2023;36(5):347–51. 37458498 10.1097/YCO.0000000000000884

[20] Badge A, Chandankhede M, Gajbe U, Bankar NJ, Bandre GR. Employment of Small-Group Discussions to Ensure the Effective Delivery of Medical Education. Cureus. 2024;16(1):e52655. 38380198 10.7759/cureus.52655 PMC10877665

[21] Kashif AW, Dagar VK, Mahato HK, Paliwal G, Datta K, Patrikar S, et al. Teaching pathology differently: Comparison between three diverse teaching learning methods. Medical journal. Armed Forces India. 2024;80(6):667–74. 10.1016/j.mjafi.2023.07.003 PMC11842911 39990529

[22] Tefera AS, Melaku EE, Urgie BM, Hassen EM, Tamene TD, Gebeyaw ED. Barriers to implementing problem-based learning at the school of medicine of Debre Berhan University, Ethiopia. BMC Med Educ. 2024;24(1):501. 38711080 10.1186/s12909-024-05252-1 PMC11075294

[23] Lim WK. Problem Based Learning in Medical Education: Handling Objections and Sustainable Implementation. Advances in medical education and practice. 2023;14:1453-60. 10.2147/AMEP.S444566 PMC10758192 38164409

