# Supplementary material for: An integrated teaching model combining PBL and medical humanities education for pathology laboratory teaching: a randomized controlled study
Source: BMC Med Educ. 2026 Jun 15;26:1349. doi: 10.1186/s12909-026-09701-x (PMC13495279; doi:10.1186/s12909-026-09701-x)
Supplement: Supplementary file 1 [file 12909_2026_9701_MOESM1_ESM.docx]

**Supplementary File S1. Student Questionnaire**

**Title:**
Student Questionnaire on Teaching Effectiveness and Satisfaction

**Introduction**

Dear student,

To comprehensively evaluate the effectiveness of this course, an anonymous survey is being conducted. This anonymous questionnaire aims to evaluate students’ perceptions of the teaching intervention in terms of learning interest, self-directed learning, communication skills, teamwork ability, professional ethics, and social responsibility in healthcare.

The results will be used solely for improving teaching quality. Your responses are very important. Please answer all questions based on your actual experience.

Thank you for your participation.

**Part I. Demographic Information**

1. **Major:** ____________________
2. **Class:** ____________________
3. **Age (years):** ____________________
4. **Sex:**
   □ Male
   □ Female

**Part II. Evaluation of Teaching Effectiveness**

**(1 = Strongly disagree, 2 = Disagree, 3 = Neutral, 4 = Agree, 5 = Strongly agree)**

**A. Learning Motivation**

| Item | Statement | 1 | 2 | 3 | 4 | 5 |
| --- | --- | --- | --- | --- | --- | --- |
| A1 | The structured teaching and guidance enhanced my motivation. |  |  |  |  |  |
| A2 | This course stimulated my interest in exploring pathology problems. |  |  |  |  |  |

**B. Self-directed Learning Ability**

| Item | Statement | 1 | 2 | 3 | 4 | 5 |
| --- | --- | --- | --- | --- | --- | --- |
| B1 | This course enhanced my initiative in acquiring knowledge. |  |  |  |  |  |
| B2 | This course improved my ability to use learning resources. |  |  |  |  |  |

**C. Communication and Expression Skills**

| Item | Statement | 1 | 2 | 3 | 4 | 5 |
| --- | --- | --- | --- | --- | --- | --- |
| C1 | My ability to ask questions and express ideas has improved. |  |  |  |  |  |
| C2 | I can write reports more clearly and logically. |  |  |  |  |  |

**D. Teamwork Ability**

| Item | Statement | 1 | 2 | 3 | 4 | 5 |
| --- | --- | --- | --- | --- | --- | --- |
| D1 | I better understand teamwork and cooperative problem-solving. |  |  |  |  |  |
| D2 | I learned to listen and communicate constructively. |  |  |  |  |  |

**E. Professional Ethics Awareness**

| Item | Statement | 1 | 2 | 3 | 4 | 5 |
| --- | --- | --- | --- | --- | --- | --- |
| E1 | This course enhanced my ethical awareness. |  |  |  |  |  |
| E2 | This course deepened my understanding of professional values. |  |  |  |  |  |

**F. Understanding of Healthcare-related Social Responsibility**

| Item | Statement | 1 | 2 | 3 | 4 | 5 |
| --- | --- | --- | --- | --- | --- | --- |
| F1 | This course strengthened my awareness of social responsibility. |  |  |  |  |  |
| F2 | This course increased attention to patients’ overall needs. |  |  |  |  |  |

**Part III. Overall Satisfaction**

How satisfied are you with the reformed teaching model of this course?

□ Very satisfied
□ Satisfied
□ Neutral
□ Dissatisfied

**Closing Statement**

Thank you again for your participation!
